# Nature, prevalence, and risk factors for self-neglect among older people: a pilot study from Vellore, South India

**DOI:** 10.1186/s12889-024-18029-4

**Published:** 2024-04-02

**Authors:** Vibisha Devaraj, Anuradha Rose, Vinod Joseph Abraham

**Affiliations:** Christian Medical College Vellore, Vellore, Tamil Nadu India

**Keywords:** Aged, Self-neglect, Depression, India, Social support, Cognition, Hygiene, Mental status and dementia tests, Social class

## Abstract

Changes in demography in developing countries haves led to new issues among older rural populations, such as self-neglect which is under researched.Self-neglect identified as poor self-care, unsafe living quarters, inadequate medical care and poor utilization of services increase the odds of morbidity and mortality.

**Methods **Our study was conducted in Kaniyambadi, a rural block in Vellore district in the state of Tamil Nadu, India. Ten villages were randomly selected. The study was conducted among people older than 60 years who were selected by random sampling using a computer-generated list. The following assessments were done: (i) A 19-item questionnaire was used to assess self-neglect, (ii) Katz index of daily living to assess functional activity, (iii) Mini Mental State Examination to evaluate cognition, (iv) Geriatric Depression Scale to identify depression, (v) Duke Social Support Index to measure social supports. Clinical data and anthropometric data were also collected. Data were entered into Epidata v3.1. All analyses were performed using SPSS v23.0.

**Results **One hundred fourteen people above 60 years of age participated. The prevalence of self-neglect was 21.1% (95% CI 14.9%—29%); about half of the elderly population (47.38%) refused to seek or follow medical advice. Lower levels of education (OR 3.678, 95% CI 1.017 – 13.301), lower social class (OR 4.455, 95% CI 1.236 – 16.050) and functional impairment (3.643, 95% CI 1.373 – 9.668) were found to be significant factors associated with self–neglect. Though prevalence of comorbidities (70%) and depression (27%) were high, there was no statistical evidence of association with self-neglect.

**Supplementary Information:**

The online version contains supplementary material available at 10.1186/s12889-024-18029-4.

## Introduction

### Background and rationale

A worldwide increase in older populations of almost double in number is expected to occur by 2050 [1]. India is also expected to see an increase in the elderly population by 9% by 2050, greatly increasing pressure for essential services for shelter, healthcare and finances for older people [2]. Help Age India has reported that 60% of older people in India have faced abuse, while the World Health Organisation expects the true numbers to be about 88%. Neglect has been reported between 33–68% of the Indian population [3, 4].

Within this silent pandemic of elder neglect lies a growing problem called self-neglect. Self-neglect defined as “the inability (intentional or nonintentional) to maintain a socially and culturally accepted standard of self-care with the potential for serious consequences to the health and well-being of the self-neglecter and perhaps even to their community” is widely accepted [4]. It has been identified by its cardinal features such as poor personal hygiene, inadequate medical care, unclean and unsafe living quarters, signs of inadequate nutrition and inattention to utility services [5]. Self-neglect has been noted to be the most common non-financial form of neglect among older people. It is frequent among those elders with certain risks such as those who live alone, have functional disabilities, have co-morbidities, especially depressive symptoms and those with poor social support [6]. The devastating medical implications of self-neglect include premature mortality and morbidity by inducing fractures, depression, dementia, malnutrition and finally death [7]. Although many studies have been undertaken in the Indian subcontinent to protect the welfare of elderly by exposing abuse and neglect, very few investigations have examined the component called self-neglect in detail. This pilot study was conducted to serve as a baseline for identifying and prioritizing further assessment needs for comprehensive care of elderly in related settings.

### Objectives

The main aim of our study was to measure the nature and prevalence of self-neglect among older people living in the Kaniyambadi block, Vellore, south India. The secondary aim of our study was to measure its associated functional, cognitive, and psychosocial risk factors.

## Methodology

### Study design

A cross-sectional community-based study was conducted in a rural region in South India.

### Study setting

The department of Community Health and Development (CHAD), Christian Medical College, Vellore, has been working in Kaniyambadi block for the past 70 years [8, 9]. This region is a geographically defined area of 127.4 km2 with 82 villages and a population of approximately 117,000. A major proportion of the population is from the lower socioeconomic strata, and agriculture and animal husbandry are the major occupations. CHAD has conducted many population surveys and maintains a computerized database of demographic details of residents. This study was conducted between September 2019 – August 2020.

### Sampling

Ten villages (out of 82) were chosen by simple random sampling. A list of all the adults aged over 60 years was retrieved from the CHAD database. Potential participants were chosen from the list by simple random sampling. The researcher (VD) visited the homes of participants selected. If a participant was not available on two occasions, the next person on the list was contacted. There were no exclusion criteria.

### Bias

To avoid selection bias, a simple random sampling method was used. To limit measurement bias, a standardized interview questionnaire and routinely calibrated equipment, such as a weighing machine and stadiometer, were used. The principal investigator (VD) was the only interviewer.

### Assessment

An interviewer administered semi-structured questionnaire was used. All the instruments in English were translated and administered in Tamil, the regional language used at the study site. The following instruments were employed to assess the participants:

(i) Proforma to assess self-neglect: A purposively designed proforma which was culturally adapted (Supplementary Material 1) was used to collect data on self-neglect. A multidisciplinary expert committee reviewed the literature [6, 10–12], extracted salient features of neglect of basic physical needs such as hygiene, environment, nutrition, health, and unsanitary and unsafe living quarters, refusal to take medications, unattended healthcare problems and poor use of medical aids. The instrument has face and content validity and was piloted for feasibility among 10 people not included in this study. The 19-item questionnaire to assess presence of self-neglect included eight observable items and 11 self-reported items. The presence of any of the above features was classified as self-neglect.
(ii) Katz Index of Independence in Activities of daily living: Katz index was developed in the 1960s by Sidney Katz is an objective way of assessing the functional status through individual’s ability to perform activities of daily living [13]. It has evolved and been simplified over more than 50 years and has been extensively used to signal functional ability of the aged in both home and clinical settings. It assesses adequacy in bathing, dressing, toileting, transferring, continence, and feeding. It has a total score of 6 and a score of 2 or less indicated complete dependence, 4 or 3 moderate dependence and 5 or 6 complete independence [14]. While there are many tools to assess functional abilities, the Katz index has been found to be useful in community settings in India due it its simplicity and adaptability even to rural settings [11].
(iii) Mini Mental State Examination: MMSE developed in 1975 is widely accepted as screening tool for assessing cognition [12]. It has been modified into a concise 11-item questionnaire that tests five areas of cognitive function: orientation, registration, attention and calculation, recall, and language [10]. The possible scores range from 0- 30 which effectively detects cognitive decline [15]. In India, MMSE has proven to positively identify those with cognitive decline with specificity of about 78% [16]. Due to translation barriers such as unavailability of validated culturally acceptable regional language phrase [17, 18] this study used a total score of 29 instead of 30, excluding a question on language (repetition of “No ifs, ands, or buts”). Participants were categorised into no cognitive decline (24–29), mild (19–23), moderate (9–18) or severe cognitive impairment (< 9). The validity after modification is expected to remain similar to other cultural modifications made in other Asian settings [18].
(iv) Geriatric Depression Scale: The Geriatric Depression Scale (GDS) developed in 1982, is a reliable screening tool for depression among older adults. It includes components relevant to depression, such as somatic complaints, cognitive complaints, motivation, future/past orientation, self-image, losses, agitation, and mood. The questions are asked in a yes/no format [19]. A concise version of GDS consisting of 15 questions with the highest correlations to depression was developed in 1986. Of the 15 items, 10 indicated the presence of depression when answered positively, while the rest indicated depression when answered negatively. It has been found to be useful even in those with mild to moderate cognitive decline. GDS has been widely accepted for usage in community settings [20]. It has been translated across the globe reliably including in the local language Tamil in a region adjacent to the study site and has a sensitivity of about 80% [21]. In our study, it was used on all except people with severe cognitive impairment. Participants were categorised based on scores into depression (> 10), probable depression (5–10) and no depression (< 5).
(v) Duke Social Support Index is an elaborate 35-item questionnaire that was developed to measure the multidimensional concept of social support. In 1993 it was abbreviated into an 11-item questionnaire to measure four dimensions of social support like network size, the frequency of social interaction, instrumental support, and subjective support. On this Likert scale, higher scores indicate higher levels of social support [22]. It has been validated for use among frail elders with non-psychiatric medical illness. The scale has good construct validity across diverse populations [23]. In this study it was used on all except people with severe cognitive impairment. The total score was 33 and elders were categorised based on mean scores as those with poor social support (≤ 16 score) and good social support (≥ 17 score).
(vi) A semi-structured questionnaire assessing the demographic details, presence of comorbidities, general examination results, and self-neglecting behaviour was used, which included questions about the basic health profile. Social class was categorized based on the BG Prasad Socioeconomic Scale [24].

### Analysis

The mean and standard deviation were obtained for continuous variables and frequency and percentage were calculated for categorical variables. Student’s t-test and chi squared tests were performed to test the significance of bivariate associations. Multivariate logistic regression was performed to adjust for confounders.

### Sample size

Sample size was calculated using the formula N = Z^2^pq/d^2^, where p is the prevalence (47.2) obtained from existing literature [14], q = (100-p) and d, precision 9.44 (20% of the prevalence). The minimum required sample size to assess self-neglect was estimated to be 112 which is considered adequate to measure the primary outcome of interest. However, the lower sample size for measuring other parameters included in the secondary outcomes is anticipated to increase in the subsequent larger study to be conducted.

## Results

One hundred thirty people were contacted, 114 of whom agreed to participate in the study while 14(10.7%) refused consent and two were unavailable. Those who refused to participate in the survey attributed this to their hesitancy to allow healthcare providers inside their homes for safety and stigma concerns as part of our study was conducted at the time of the emergence of the Covid 19 pandemic. There was no statistically significant difference in age and gender between those who participated in the study and those who did not.

Table 1 shows the sociodemographic and clinical characteristics of the participants. The majority of participants (57.9%) were less than 70 years of age, were women, were literate, were currently unemployed, were from the middle socio-economic class and lived with their families. Most of the participants (70.2%) had medical comorbidities and also had significant disabilities. A small but significant proportion of the population [21.1% (95% CI 14.9%—29%)] had self-neglect, poor social support, severe cognitive decline, significant depression, and impairments in activities of daily living.

**Table 1:** Sociodemographic and clinical characteristics of the sample (n-114)

Characteristic	Descriptive statistics
	**Mean (SD)**
Age in years	69.33 (8.014)
	**Frequency n (%)**
Gender- Male	50 (43.9)
Marital status -currently married	70 (61.4)
Literacy-Illiterate	46 (40.4)
Employment status- Unemployed	57 (50.0)
Social class- lower	47 (41.2)
Living alone	13 (11.4)
Comorbidities -present	80 (70.2)
Diabetes mellitus -present	37 (32.5)
Hypertension -present	42 (36.8)
Disability -present	53 (46.5)
Vision impairment- present	27 (23.7)
Hearing impairment- present	13 (11.4)
Musculoskeletal problems- present	22 (19.3)
Self -neglect-present	24 (21.1)
Severe cognitive decline-present	3 (2.6)
Depression- present	11 (10)
Activities of daily living impairment	5 (4.4)
Social support—poor	7 (6.3)

Table 2 reports the factors associated with self -neglect. Self-neglect was significantly associated with lower education and socioeconomic status, disability, hearing loss and impairment in activities of daily living. Among those with low and middle socio-economic status, after adjusting for other factors, poor social support (AOR -6.11, *p*-value – 0.023) was found to be significantly associated with the presence of self-neglect.

**Table 2:** Factors associated with self-neglect

Characteristics	Self-neglect		Bivariate Statistics	
	**Present n (%)**	**Absent n (%)**	**OR (95% CI)**	***p* value**
Gender-male	7(14.0)	43(86.0)	0.450(0.170–1.190)	0.103
Age ≥ 67years	16(28.1)	41(71.9)	2.390(0.929 – 6.148)	0.066
**Education ≤ 7th grade**	**21(26.3)**	**59(73.8)**	**3.678(1.017 – 13.301)**	**0.037**
Marital status—Widowed	13(29.5)	31(70.5)	2.249(0.943 – 5.605)	0.078
Number of people living at home ≤ 3	12(21.1)	45(78.9)	1.00(0.406 – 2.461)	1.000
**Social class -Low**	**21(27.6)**	**55(72.4)**	**4.455(1.236 – 16.050)**	**0.015**
Comorbidities- present	20(25)	60(75)	2.50(0.784 – 7.971)	0.113
**Disability- present**	**17(32.1)**	**36(67.9)**	**3.643(1.373 – 9.668)**	**0.007**
**Hearing disability-present**	**6(46.2)**	**7(53.8)**	**3.952(1.186 – 13.170)**	**0.029**
**Activities of daily living- Impaired**	**5(100)**	**0(0)**		** < 0.001**
Depression- present	10(24.4)	31(75.6)	1.730(0.662 – 4.521)	0.260
Cognitive decline—present	11(28.9)	27(71.1)	1.974(0.786 – 4.958)	0.144
Social support- Poor	2(28.6)	5(71.4)	1.789(0.322 – 9.930)	0.615

## Discussion

This study measured the prevalence and associated factors of self-neglect among older people living in rural South India. The baseline characteristics of the study population was comparable to other older people in India thus demonstrating generalizability to senior citizens in rural regions across the country. The mean age of studied population was 69.3, however the mode was 65 reflecting the life expectancy of about 67 in India [25]. Since literacy mission in India was launched only in 1988 the literacy levels among elderly have been about 42.7% in the country. However, literacy rates in Tamil Nadu and Vellore have been higher (43–56%) which parallels with the study findings (59.6%) [26–28]. This could be attributed to the revolutionary government schemes established in 1950s in the state [29]. This study showed close to half of the elders belonging to lower or lower middle social class similar to other areas(38.2- 67.3%) [30, 31]. India has been recording an increase in women employment, yet currently only 27% of Indian women are in the labour force and as expected in the given population, nearly 3/4th of the women were homemakers [32].

The study population had a much higher number of widow/er (38.4% v 26.0%) [33], and more medical comorbidities (70.0% v 53.1%) [34]. Compared to those of other older populations in the country, the incidence of musculoskeletal disability (19.3% v 56%) [35], obesity (35% v 50–70%) [36, 37], pallor (20.2% v up to 68%) [38, 39], glossitis (9.6 v up to 37%) [38, 39], depression (27% v 34.4%) [40], functional impairment (4.4% v 30%) [41] and living alone (10% v 28.5%) [42] were much lower. Rates of hypertension, diabetes, ophthalmic disabilities, and cognitive decline were similar [35, 37, 40, 43–46].

The prevalence of self-neglect in the study population was found to be 21.2% (95% CI 14.9% -29%) which is lower than the prevalence reported in other older populations. Most of the participants with self-neglect had more than one self-neglecting feature (71%). While hoarding, untended home surroundings, unsanitary conditions, and inadequate utility were the common phenotypes of self-neglect noted in Western countries [47], negligence in seeking medical care (50%) and refusal to take treatment (37.5%) were common in this study population. Self-neglect among elderly is associated with multiple co-morbidities and unfortunately many self-neglecting elders refuse medical care due to denial, feeling ashamed of needing help and fear of losing independence [48, 49].

Poor personal care (e.g., unkempt hair or nails, wearing soiled clothing) was the common feature in this setting. About 5% of them were found to be living in unsanitary conditions, smeared with urine or feaces and three of them had insect infestation such as ants and maggots on them. It was also observed that five of them were living in conditions which could cause accidental falls.

The majority of the participants, even those who were living alone were found to have good social support in this study which differs from older populations in other settings [50, 51]. The cultural attributes of the rural population and their sense of community dwelling in the regions explain these differences.

Self-neglect was significantly associated with lower education, lower social class impaired functional activity, people with disability especially among those with hearing impairment. There was significant association between poor social support and self-neglect among those with lower income after adjusting for other factors.

One of the major strengths of our study is that, to the bet of the author’s knowledge, this is the first study analysing the prevalence of self-neglect among older rural South Indians. The data used in our study was obtained directly from the participants without the use of secondary sources of information. As the primary researcher was familiar with the study population and language, it was easier to establish good rapport with the participants which enabled us to obtain reliable information including sensitive issues such as family support. However, our study findings need to be interpreted in the context of the following limitations. The sample size was powered to assess self-neglect prevalence, however given the study setting, more number of subjects might be needed to assess association with factors such as social support, impaired activities of daily living and social class. Medical chart review of the participants could not be performed which might have provided additional information on complications of self-neglect. Participants identified with self-neglect could not be followed up due to the COVID-19 pandemic.

Large population studies are required to study associations between self-neglect and related factors in rural context. The striking high prevalence of co-morbidities among the elders with possible risk of under estimation in this population warrants requirement of strengthening preventive health measures.

## Conclusion

Self-neglect is a rapidly emerging yet under-researched issue particularly in the rural regions of South India. Our study has identified that one in five older rural South Indians have some features of self-neglect. While there are similarities and differences between the study population and older people living in other parts of India and Asia, the fact that nearly half of the study population refused to seek medical interventions, suggests the need for community monitoring of the health of older people living in India. The projected increase in older people in India over the next few decades, demands increased community and health support so that older people can live independently with dignity and have a better quality of life.

### Supplementary Information

**Supplementary Materials 1.**

## Data Availability

The datasets used and analysed during the current study are available from the corresponding author on reasonable request.

## Abbreviation

CHAD: Department of Community Health And Development Vellore
