# Supplementary material for: Nature, prevalence, and risk factors for self-neglect among older people: a pilot study from Vellore, South India
Source: BMC Public Health. 2024 Apr 2;24:948. doi: 10.1186/s12889-024-18029-4 (PMC10986042; doi:10.1186/s12889-024-18029-4)
Supplement: Supplementary file 1 — Supplementary Materials 1. [file 12889_2024_18029_MOESM1_ESM.docx]

**Supplementary Material 1**

**Questionnaire:**

S. No. Name: Age: Gender:

Marital status: (Single/Married/Widowed/Separated) Education: Occupation:

Address:

Total number of family members: Per capita monthly income:

SELF NEGLECT QUESTIONNAIRE:

1. Is there dirty, faecal and/or urinary odour on the person? – Y/N
2. Is there unkempt (apart from religious reasons) and dirty hair and/or uncut dirty nails in the person? – Y/N
3. Is there any insect infestation on the person? – Y/N
4. Is the person wearing unclean clothing? – Y/N
5. Is there any rash, bruises and/or sores for which the person hasn’t sought treatment? – Y/N
6. Is there any skin Infection for which the person hasn’t sought treatment? – Y/N
7. Does she/he have difficulty in hearing and has hearing aids but is not using? -Y/N
8. Has the person been advised to use dentures, has it but is not using? – Y/N
9. Does he/she have any prosthesis or walking aid that is currently required but is not using it? -Y/N
10. Does the person have urinary incontinence for which treatment hasn’t been sought? – Y/N
11. Does the person have constipation or faecal incontinence for which treatment hasn’t been sought? – Y/N
12. Are the floors or walls stained with faecal matter or foul smelling? – Y/N
13. Is there presence of urine or faeces in the living area? – Y/N
14. Are there unsafe live wires, or leaking pipes in the house?
15. Are there falling roofs/walls or objects scattered or arranged in a manner that can cause tripping over? – Y/N
16. Does the person have any illness or told to have an illness but hasn’t sought treatment? – Y/N
17. Has the person been asked to see a doctor for check-up or follow-up but hasn’t visited as prescribed? – Y/N
18. Was the person advised any medicine but is taking more than prescribed or taking unprescribed medication? (to care-giver) – Y/N
19. Is the person refusing to take prescribed medications? (to care-giver) – Y/N

GENERAL EXAMINATION:

Height (in cm)- Weight (in Kg) – BMI

Icterus/Cyanosis/Clubbing/Lymphadenopathy/Edema BP(mmHg) – Dehydration– Y/N

Signs of malnutrition: Yes/No

(Pallor, Bitot’s spots, xerophthalmia, impaired vision at night , bleeding gums, angular stomatitis, chelitis , apthous ulcers, glossitis, Koilonychia, Platynychia ) –

MORBIDITY: (Yes/No)

- 1. Diabetes -
  2. Hypertension -
  3. COPD/Asthma -
  4. Old CVA-
  5. IHD-
  6. Seizure Disorder-
  7. Disabled – ophthal/hearing/MSK/mental
  8. Others -
